# A Two-Parameter Fractional Tsallis Decision Tree

**DOI:** 10.3390/e24050572

**Published:** 2022-04-19

**Authors:** Jazmín S. De la Cruz-García, Juan Bory-Reyes, Aldo Ramirez-Arellano

**Affiliations:** 1 SEPI-UPIICSA, Instituto Politécnico Nacional, Mexico City C.P. 08400, Mexico; susdcg@gmail.com; 2 SEPI-ESIME-ZACATENCO, Instituto Politécnico Nacional, Mexico City C.P. 07738, Mexico; juanboryreyes@yahho.com

**Keywords:** decision trees, complex networks, two-parameter Tsallis entropy, Gini index

## Abstract

Decision trees are decision support data mining tools that create, as the name suggests, a tree-like model. The classical C4.5 decision tree, based on the Shannon entropy, is a simple algorithm to calculate the gain ratio and then split the attributes based on this entropy measure. Tsallis and Renyi entropies (instead of Shannon) can be employed to generate a decision tree with better results. In practice, the entropic index parameter of these entropies is tuned to outperform the classical decision trees. However, this process is carried out by testing a range of values for a given database, which is time-consuming and unfeasible for massive data. This paper introduces a decision tree based on a two-parameter fractional Tsallis entropy. We propose a constructionist approach to the representation of databases as complex networks that enable us an efficient computation of the parameters of this entropy using the box-covering algorithm and renormalization of the complex network. The experimental results support the conclusion that the two-parameter fractional Tsallis entropy is a more sensitive measure than parametric Renyi, Tsallis, and Gini index precedents for a decision tree classifier.

## 1. Introduction

Entropy is a measure of the unpredictability of the state in physical systems that would be needed to specify the degree of disorder in full micro-structure of them. Claude Elwood Shannon [1] defined a measure of entropy to measure the amount of information in a digital system in the context of theory communication that has been applied in a variety of fields such as information theory, complex networks, and data mining techniques.

The most widely used form of the Shannon entropy is given by

(1) $$I=\lim_{t\to -1}\frac{d}{dt}\sum_{i=1}^{N}p_{i}^{-t}=-\sum_{i=1}^{N}p_{i}\ln p_{i},$$

where *N* is the number of possibilities $p_{i}$ and $\sum_{k=1}^{n}p_{k}=1$.

Two celebrated generalizations of Shannon entropy are Renyi [2] and Tsallis entropies [3]. Alfred Renyi proposed a universal formula to define a family of entropy measures given by the expression [2]

(2) $$I_{q}^{R}=\frac{1}{1-q}\sum_{i=1}^{N}p_{i}^{q},$$

where *q* denotes the order of moments.

Constantino Tsallis proposed the q-logarithm defined by

(3) $$ln_{q}\left(x\right)=\frac{x^{1-q}-1}{1-q},$$

to introduce a physical entropy given by [3]

(4) $$I_{q}^{T}=-\sum_{i=1}^{N}p_{i}ln_{q}p_{i}=\frac{1}{q-1}\left(1-\sum_{i=1}^{N}p_{i}^{q}\right).$$

Tsallis entropy could be rewritten [4,5,6] as

(5) $$I_{q}^{T}=\lim_{t\to -1}D_{q}^{t}\sum_{i=1}^{N}p_{i}^{-t},$$

where $D_{q}^{t}$ of a function *f* given by $D_{q}^{t}f\left(t\right)=\frac{f(qt)-f(t)}{(q-1)t}$, t≠0, stands for the Jackson [7] q-derivative, to reflect that it is an extension of Shannon entropy.

Renyi and Tsallis entropy measures depend on the parameter *q*, which describes their deviations from the standard Shannon entropy. Both entropies converge to Shannon entropy in the limit q→1. For complex network applications [8] and data mining techniques [9,10,11,12,13,14,15,16,17], the parameter *q* varies into a range of values. On the other hand, the computation of the entropic index *q* of the Tsallis entropy was implemented for physics applications in [18,19,20,21,22,23,24,25].

Shannon and Tsallis entropies can be obtained by the action of standard derivative or q-derivative, respectively, to the same generating function $\sum_{i=1}^{N}p_{i}^{-t}$ with respect to variable *t* and then letting t→−1. This approach can be used to reveal different entropy measures based on the actions of appropriate fractional order differentiation operators [26,27,28,29,30,31,32].

The major goal of this work is to introduce a new decision tree based on a two-parameter fractional Tsallis entropy. This new kind of tree is tested on twelve databases for a classification task. The structure of the paper is as follows. Section 2 focuses attention on the notion of two-parameter fractional Tsallis entropy. In Section 3, two-parameter fractional Tsallis decision trees and a constructionist approach to the representation of the databases as a complex network are introduced. The basic facts on the box-covering algorithm of a complex network are reviewed. Finally, we compute an approximation set of parameters *q*, β, and α of the two-parameter fractional Tsallis entropy. Section 4 is concerned with the testing of two-parameter fractional Tsallis decision trees on twelve databases. Next, the approximations of *q* values are tested on Renyi and Tsallis entropies. Discussion of the findings of this study and concluding remarks are offered in Section 5.

## 2. Two-Parameter Fractional Tsallis Entropy

Based on the actions of fractional order differentiation operators, several entropy measures of fractional order are introduced in [26,27,28,29,30,33,34,35,36,37,38,39,40,41,42,43]. Following this approach in [22], the two-parameter fractional Tsallis entropy is introduced by merging two typical examples of fractional entropies.

The first fractional entropy of order δ∈(0,1] is introduced as

(6) $$I_{\delta }^{1}=\lim_{t\to -1}\frac{d^{\delta }}{dt}\sum_{i=1}^{N}p_{i}^{-t}=-\sum_{i=1}^{N}p_{i}^{\delta }\ln p_{i},$$

and the second one by

(7) $$I_{\delta }^{2}=\lim_{t\to -1}\frac{d}{dt}\left({}_{-\infty }D_{t}^{\delta -1}\sum_{i=1}^{N}e^{-t\ln p_{i}}\right)=\sum_{i=1}^{N}p_{i}{\left(-\ln p_{i}\right)}^{\delta },where$$

(8) $$\frac{d^{\delta }f\left(t\right)}{dt}=\lim_{h\to 0}\frac{f^{\delta }\left(t+h\right)-f^{\delta }\left(t\right)}{{\left(t+h\right)}^{\delta }-h^{\delta }}$$

and

(9) $${}_{-\infty }D_{t}^{\delta -1}f\left(t\right)=\frac{1}{\Gamma (1-\delta )}\int_{-\infty }^{t}\frac{f(t^{\prime })}{{\left(t-t^{\prime }\right)}^{\delta }}dt^{\prime },$$

where Γ denotes the gamma function.

Combining (6) with (7) yields a two-parameter fractional relative entropy as follows [31]:

(10) $$I_{\beta }^{\alpha }=\sum_{i=1}^{N}p_{i}^{\alpha }{\left(-\ln p_{i}\right)}^{\beta },$$

for 0<α,β. The entropy (10) reduces to (6) when β→1 and reduces to (7) when α→1, yielding the Shannon entropy when both parameters approach 1.

Analogously, two extra-parameter-dependent Tsallis entropies are introduced [22]:

(11) $$T_{\delta ,q}^{1}=-\sum_{i=1}^{N}p_{i}^{\delta }ln_{q}p_{i}.$$

and

(12) $$T_{\delta ,q}^{2}=\sum_{i=1}^{N}p_{i}{\left(-\ln_{q}p_{i}\right)}^{\delta }.$$

Combining theses entropies and motivated by (10), we obtain the following two-parameter fractional Tsallis entropy [22]:

(13) $$T_{q}^{\alpha ,\beta }=\sum_{i=1}^{N}p_{i}^{\alpha }{\left(-\ln_{q}p_{i}\right)}^{\beta },$$

for 0<α,β.

Note that Tsallis entropy is recovered when $\lim_{\alpha ,\beta \to 1}T_{q}^{\alpha ,\beta }$. This implies that the non-extensibility of $T_{q}$ [44] forces $T_{q}^{\alpha ,\beta }$ to be so.

## 3. Parametric Decision Trees

**Figure 1:**
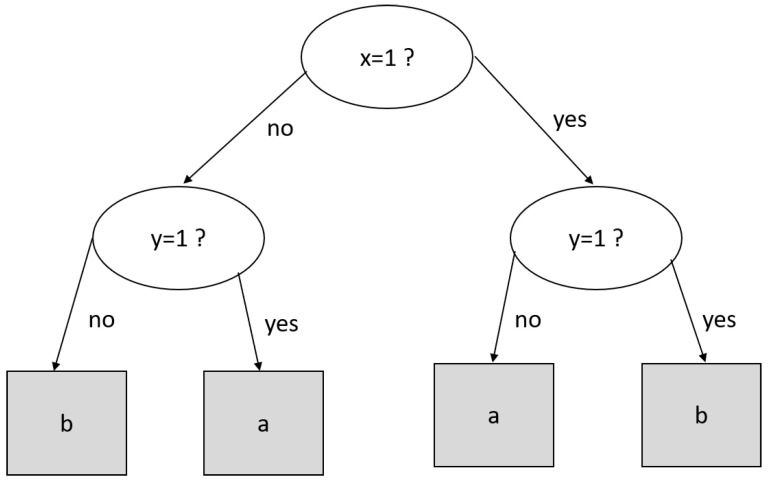
A decision tree to the classification task.

A decision tree is a supervised data mining technique that creates a tree-like structure, where the non-leaf node tests a given attribute [45]. The outcome gives us the path to reach a leaf node, where the classification label is found. For example, let (*x* = 3, *y* = 1) be a tuple to be classified by the decision tree of Figure 1. If we test x=1, we must follow the left path to reach y=1 and finally arrive at the leaf node with the classification label “*a*”.

In general, the cornerstone of the construction process of decision trees is the evaluation of all attributes to find the best node and the best split condition on this node to classify the tuple with the lower error rate. This evaluation is carried out by information gain on each attribute *a* [45]:

(14) $$G_{a}=I\left(D\right)-I_{c}\left(D\right),$$

where I(D) is the entropy of the database after being partitioned by the condition *c* of a given attribute *a* and $I_{c}\left(D\right)$ is the entropy induced by *c*. The tree’s construction needs to evaluate several partition conditions *c* on all attributes of the database, then chooses the pair of attribute–condition with the highest value. Once a pair is chosen, the process evaluates the partitioned database recursively using a different attribute–condition. The reader is referred to [45] for details on decision tree construction and computation of (14).

### 3.1. Renyi and Tsallis Decision Trees

In classical decision trees, *I* in (14) denotes Shannon entropy; however, other entropies such as Renyi or Tsallis can replace it. Thus, (14) can be written using Renyi entropy (2) as

(15) $$G_{R,a}=I_{q}^{R}\left(D\right)-I_{q,c}^{R}\left(D\right),$$

and using Tsllis entropy (4) as follows:

(16) $$G_{T,a}=I_{q}^{T}\left(D\right)-I_{q,c}^{T}\left(D\right).$$

The parametric decision trees generated by (15) or (16) have been studied in [9,10,11,12,13,14].

### 3.2. Two-Parameter Fractional Tsallis Decision Tree

Following a similar fashion, a two-parameter fractional decision tree can be induced by the information gain obtained by rewritten (14) using (13):

(17) $$G_{T_{q}^{\alpha ,\beta },a}=T_{q}^{\alpha ,\beta }\left(D\right)-T_{q,c}^{\alpha ,\beta }\left(D\right),$$

An alternative informativeness measure for constructing decision trees is the Gini index, or Gini coefficient, which is calculated by

(18) $$Gini=1-\sum_{i=1}^{N}p_{i}^{2}.$$

The Gini index can be deduced from Tsallis entropy (4) using q=2 [14]. On the other hand, the two-parameter fractional Tsallis entropy with q=2, α=1, β=1 reduces to the Gini index. Hence, Gini decision trees are a particular case of both Tsallis and two-parameter fractional Tsallis trees.

The main issue with Renyi and Tsallis decision trees is the estimation of q-value to obtain a better classification than the one produced by the classical decision trees. Trial and error is the accepted approach for this purpose. It consists of testing several values in a given interval, usually [−10,10], and comparing the classification rates. This approach becomes unfeasible in two-parameter fractional Tsallis decision trees as it is needed to tune *q*, α, and β. A representation of a database as a complex network is introduced to face this issue. This representation lets us compute α and β following the approach in [22], which is the basis for determining the fractional decision tree parameters.

### 3.3. Network’s Construction

A network is a powerful tool to model the relationships among entities or parts of a system. When those relationships are complex, i.e., properties that cannot be found by examining single components, something emerges that is called a complex network. Thus, networks as a skeleton of complex systems [46] have attracted considerable attention in different areas of science [47,48,49,50,51]. Following this approach, a representation of the relationships among attributes (system entities) of a database (system) as a network is obtained.

**Figure 2:**
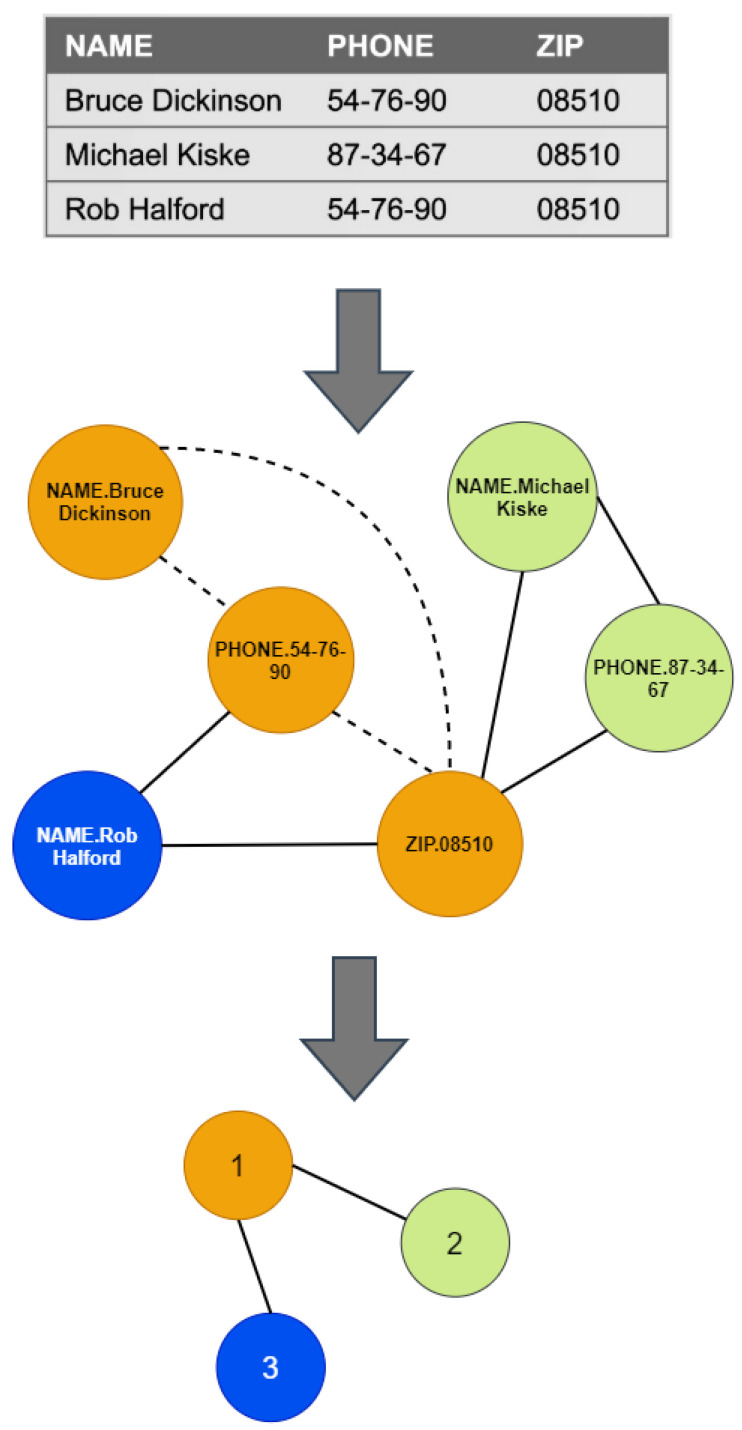
Network construction from a database. The nodes in the same color belong to the same box for l=2.

The attribute’s name will be concatenated before the value of a given row to distinguish the same value that might appear on different attributes. Consider the first record of the database shown on the top of Figure 2. The first node will be NAME.BruceDickinson, the second node will be PHONE.54−76−90, and the third node will be ZIP.08510. These nodes belong to the same record, so they must be connected; see dotted lines of the network in the middle of Figure 2. We next consider the second record; the nodes Name.MichaelKiske and PHONE.87−34−67 will be added to the network. Note that the node ZIP.08510 was added in the previous step. We may now add the links between these three nodes. This procedure is repeated for each record in the database.

The outcome is a complex network that exhibits non-simple topological features [52], which cannot be predicted by analyzing single nodes as occurs in random graphs or lattices [53].

### 3.4. Computation of Two-Parameter Fractional Tsallis Decision Tree Parameters

By the technique introduced in [22], the parameters α and β—on the network representation of the database—of the two-parameter fractional Tsallis decision tree are defined to be

(19) $$\alpha_{l,i}^{1}=1+\frac{|G_{i}|innerdeg\left(G_{i}\right)}{n\sum_{i=1}^{N_{b}}innerdeg\left(G_{i}\right)},$$

(20) $$\alpha_{l,i}^{2}=1-\frac{|G_{i}|innerdeg\left(G_{i}\right)}{n\sum_{i=1}^{N_{b}}innerdeg\left(G_{i}\right)},$$

where $|G_{i}|$ is the number of nodes in the box $G_{i}$ obtained by the box-covering algorithm [54], *n* is the number of nodes of the network, and $innerdeg(G_{i})$ is the average degree of the nodes of the $G_{i}$ box. Similarly, two values of β are computed as follows [22]:

(21) $$\beta_{l,i}^{1}=1+\frac{outerdeg(G_{i})l}{\Delta \sum_{i=1}^{N_{b}}outerdeg\left(G_{i}\right)},$$

(22) $$\beta_{l,i}^{2}=1-\frac{outerdeg(G_{i})l}{\Delta \sum_{i=1}^{N_{b}}outerdeg\left(G_{i}\right)},$$

where l>2 is the diameter of the box $G_{i}$, Δ is the diameter of the network, and $outerdeg(G_{i})$ is the number of links among the boxes $G_{i}$. The computation of innerdeg and outerdeg will be explained later.

Inspired by the right-hand term of (19) and (20) (named $\alpha^{\prime }$) with the fact that $\delta =\frac{lN_{b}\left(l\right)}{\Delta \sum_{l=2}^{\Delta }N_{b}\left(l\right)}$ is a normalized measure of the number of boxes to cover the network [20], an approximation of the q-value for the two-parameter fractional decision tree is introduced:

(23) $$q_{\alpha^{\prime }}=\frac{\delta }{\alpha^{\prime }}=\frac{nlN_{b}\left(l\right)\sum_{i=1}^{N_{b}}innerdeg\left(G_{i}\right)}{\Delta |G_{i}|innerdeg\left(G_{i}\right)\sum_{l=2}^{\Delta }N_{b}\left(l\right)}.$$

Similarly, from the right hand of (21) and (22) (named $\beta^{\prime }$), a second approximation of the q-value is given by

(24) $$q_{\beta^{\prime }}=\frac{\delta }{\beta^{\prime }}=\frac{N_{b}\left(l\right)\sum_{i=1}^{N_{b}}outerdeg\left(G_{i}\right)}{outerdeg\left(G_{i}\right)\sum_{l=2}^{\Delta }N_{b}\left(l\right)},$$

where $N_{b}\left(l\right)$ is the minimum number of boxes of diameter *l* to cover the network, *n*, Δ, $N_{b}\left(l\right)$, $|G_{i}|$.

**Figure 3:**
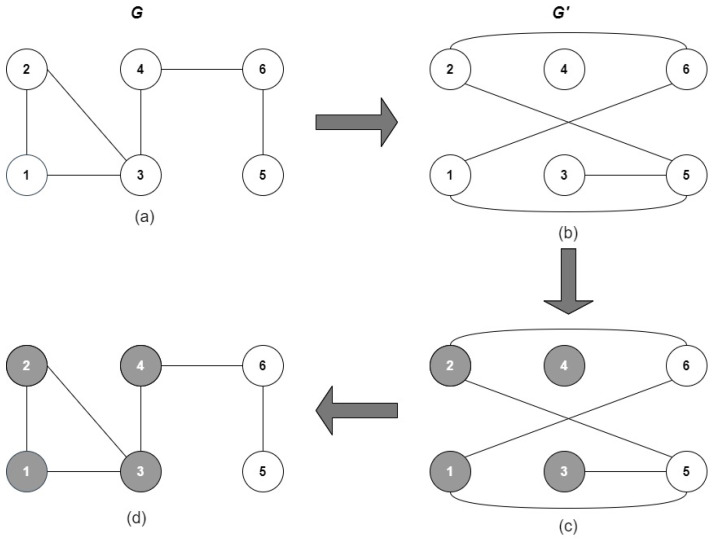
Box covering of a network for l=3. (**a**) Original network. (**b**) Dual network. (**c**) Colouring process. (**d**) Mapping colours to the original network.

The process to compute the minimum number of boxes $N_{b}$ of diameter *l* to cover the network *G* is shown in Figure 3. A dual network ($G^{\prime }$) is created only with the nodes of the original network, Figure 3b. Then, the links in $G^{\prime }$ are added following the rule: two nodes *i*, *j*, in the dual network, are connected if the distance between $l_{ij}$ is greater than or equal to *l*. In our example, l=3, and node one is selected to start. Node one will be connected in $G^{\prime }$ with nodes five and six since their distance is four and three. The procedure is repeated with the remaining nodes to obtain the dual network shown in Figure 3b. Next, the nodes will be colored as follows: two directly connected nodes in $G^{\prime }$ must not have the same color. Finally, the nodes colored in $G^{\prime }$ are mapped to *G*; see Figure 3c. The minimum number of boxes to cover the network given *l* equals the number of colors in *G*. In addition, the nodes in the same color belong to the same box. In practice, l=[1,Δ]; thus, $N_{b}\left(l\right)$ of the example are shown in Table 1. For details of the box-covering algorithm, the reader is referred to [54].

**Figure 4:**
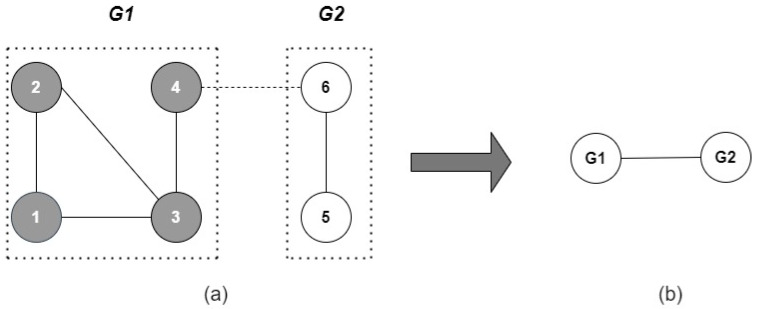
Renormalization of a network. (**a**) Grouping nodes into boxes. (**b**) Converting boxes into supernodes.

Now, we are ready to compute innerdeg. Two boxes were found following the previous example for for l=3; see Figure 4a. The $innerdeg(G_{1})=2$ is the average link per node between the nodes of this box; for this reason, the link between nodes four and six is omitted in this computation. Similarly, $innerdeg(G_{2})=1$. The outerdeg is the degree of each node of the renormalized network; see the network of Figure 4b.

In our example, $outerdeg\left(G_{1}\right)=outerdeg\left(G_{2}\right)=1$. The renormalization converts each box into a super node, preserving the connections between boxes. On the other hand, it is known that $N_{b}\left(1\right)=n,$ and $N_{b}\left(\Delta +1\right)=1$; in the first case, each box contains a node, and in the second one, there is one box to cover the network that contains all nodes. For this reason, the innerdeg and outerdeg are not defined for l=1 and l=Δ+1, respectively. This force to l=[2,Δ] as was stated in (19)–(24). Additionally, note that the right hand of (19) and (20) ($\alpha^{\prime }$), (21) and (22) ($\beta^{\prime }$) are “pseudo matrices”, where each row has $N_{b}\left(l\right)$ values; see Table 1. Consequently, $q_{\alpha^{\prime }}$ and $q_{\beta^{\prime }}$ are also “pseudo matrices”.

The network represents the relationships between attribute-value (nodes) of each record and the relationships between different database records. For example, the dotted lines in Figure 2 show the relationships between the first record’s attribute value. Links of the node ZIP.08510 are the relationships between the three records, and the links of PHONE.54-76-90 are the relationships between the first and third one. The box-covering algorithm groups these relationships into boxes (network records). The network in the middle of Figure 2 shows that the three boxes (in orange, green, and blue) coincide with the number of records in the database. However, the attribute value of each box does not coincide entirely with records in the database since box-covering finds the minimum number of boxes with the maximum number of attributes where the boxes are mutually exclusive.

The nodes in each box (network record) are enough to differentiate the records in the database. For example, the first network record consists of name, phone, and zip values (nodes in orange). The second record in the database can be differentiated from the first by its name and phone (values of those attributes are the second network record in green). The third one can be distinguished from the two others by its name (the third network record in blue). The cost of differentiating the first network record (measured by innerdeg) is the highest; meanwhile, the lowest is for the third. Thus, $\alpha^{\prime }$ measures the local differentiation cost for the network records.

On the other hand, $\beta^{\prime }$ measures the global differentiation cost (by outerdeg). For example, the global cost for the first network record is two, and one for the second and third; see the renormalized network (at the bottom of Figure 2). It means that the first network record needs to be differentiated from two network records, and the second and third only need to be distinguished from the first. Note that $\alpha^{\prime }$, $\beta^{\prime }$ for a given *l* relies on the topology network that captures the relationships of the records and their values. Finally, $q_{\alpha^{\prime }}$ is the ratio between network records (normalized number of boxes δ) and the local differentiation cost; meanwhile, $q_{\beta^{\prime }}$ is the ratio between network records and the global differentiation cost.

## 4. Methodology

Twelve databases (biological, technological, and social disciplines) from the UCI repository [55] were managed in the experiments; see Table 2. Their number of records, attributes, and classes are representative. Once a network was obtained from the database, *q*, α, and β parameters of the fractional Tsallis decision were approximated by the following four sets: $(<q_{\alpha^{\prime }}>$, $<\alpha^{1}>$, $<\beta^{1}>)$, $(<q_{\alpha^{\prime }}>$, $<\alpha^{2}>$, $<\beta^{2}>)$, $(<q_{\beta^{\prime }}>$, $<\alpha^{1}>$, $<\beta^{1}>)$, $(<q_{\beta^{\prime }}>$, $<\alpha^{2}>$, $<\beta^{2}>)$, where <> means the average value of the pseudo matrices obtained by (19)–(24).

**Figure 5:**
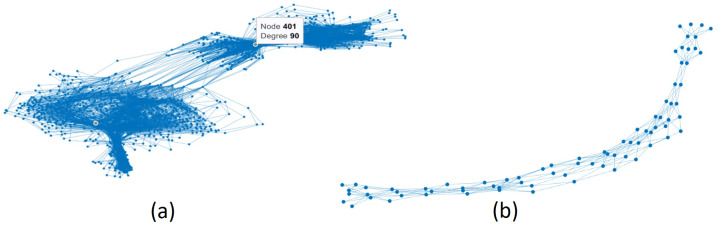
The networks from (**a**) non-discretized and (**b**) discretized vehicle database.

The network can be obtained from a raw database or after being discretized. Since the classification—measured by the area under receiver operating characteristic curve (AUROC) and Matthews correlation coefficient (MCC)—was better using the approximations computed on the networks from discretized databases, these approximations are only reported. The attribute discretization of a database can be found in [56]. The discretization technique is unsupervised and uses equal-frequency binning. The discretized databases were only used to obtain the networks so that the classification task was carried out using the original databases. The networks obtained from the discretized and non-discretized databases turned out to be different; see Figure 5.

The classification task was performed by classical, Renyi, Tsallis, Gini, and the two-parameter fractional Tsallis decisions trees on each database. We used a 10-fold cross-validation repeated ten times to calculate the AUROC and MCC. The best value of the AUROC and MCC, produced by one of the four sets of parameters—used to approximate *q*, α, and β—of fractional Tsallis decisions trees, was chosen and compared with the classical and Gini decision trees. In the same way, $q_{\alpha^{\prime }}$ or $q_{\beta^{\prime }}$ was chosen for the *q* parameter of Renyi and Tsallis trees. Then, their AUROCs and MCCs were compared with those of the classical trees. It is known that decision trees could produce non-normal distributed AUROC and MCC measures [57]. Hence, the normality was verified by the Kolmogorov–Smirnov test. These measures were compared using a *T* or a *U* Mann–Whitney test, according to their normality [10,57,58,59].

## 5. Applications

The approximations of *q*, α, and β parameters computed on discretized databases are shown in Table 3. Table 4 shows the AUROC and MCC of classical and two-parameter fractional Tsallis decisions trees and the result of the statistical compassion. In addition, the values of the parameters of fractional Tsallis decisions trees are reported.

The two-parameter fractional Tsallis decision tree outperforms the AUROC and MCC of the classical trees for eight databases. The statistical result of both measures disagrees with Car and Haberman. The AUROC of the two-parameter fractional Tsallis tree was equal to the classical trees for Car, Image, Vehicle, and Yeast; meanwhile, for Haberman, Image, Vehicle, and Yeast, the MCC of both trees showed no difference.

Tsallis entropy is a non-extensive measure [60] as well as a two-parameter fractional Tsallis entropy [22]. On the contrary, Shannon entropy is extensive. The super-extensive property is given by q<1, and sub-extensive property by q>1. Note that the approximations of the *q* parameter for all the databases, see Table 3, are <1 except for Yeast. Thus, they can be considered candidates for being named super-extensive databases. We say that a database is super-extensive if q<1 and its value produces a better classification (AUROC, MCC, or another measure) than the classical trees (based on Shannon entropy). Similarly, a database is sub-extensive if q>1 and its value produces a better classification. Otherwise, the database is extensive since, in this case, the Shannon entropy (the cornerstone of classical trees) is a less complex measure than the two-parameter fractional Tsallis entropy; hence Shannon entropy must be preferred. The two-parameter fractional Tsallis trees produce classifications equal to or better than the classical trees. Following those conditions, based on MCC, Breast Cancer, Car, Cmc, Glass, Hayes, Letter, Scale, and Wine are super-extensive. Meanwhile, Haberman, Image, Vehicle, and Yeast can be classified as extensive.

The AUROC and MCC of Renyi and Tsallis decision trees are compared with the baseline of the classical ones. The $q_{\alpha^{\prime }}$ and $q_{\beta^{\prime }}$ were tested as the entropic index of both parametric decision trees. The parameters of Renyi ($q_{r}$) and Tsallis ($q_{t}$) that produce the better AUROCs and MCCs are reported in Table 5. The result shows that the AUROC of Renyi trees was better for Breast Cancer, Glass, Letter, and Yeast and worse for Cmc and Haberman than classical trees. The results are quite similar for MCC, where Car’s classification outperforms the classical tree classification. On the contrary, the MCC of the Vehicle database was statistically less than that of the classical tree. The Tsallis AUROCs were better for Cmc, Glass, Haberman, Hayes, and Wine and worse for Yeast than those of classical trees. Additionally, the MCCs of Car, Cmc, Glass, and Scale were higher, and lower for Yeast, than the classical trees’ MCCs. Based on MCC, Car, Cmc, Glass, and Scale are super-extensive, which is a subset of the classification obtained by two-parameter fractional Tsallis.

Finally, the Gini and the two-parameter fractional Tsallis decisions trees are compared using AUROC and MCC. The results are shown in Table 6. These results indicate that two-parameter fractional Tsallis trees outperform AUROC of Gini trees in six databases, and MCC in ten. It underpins that Gini trees are a particular case of two-parameter fractional Tsallis trees with q=2. In summarizing, two-parameter fractional Tsallis trees have better classifications than classical and Gini trees.

## 6. Conclusions

This paper introduces two-parameter fractional Tsallis decision trees underpinned by fractional-order entropies. The three parameters of this new decision tree need to be tuned to produce better classifications than the classical ones. The trial and error approach is the standard method to adjust the entropic index for Renyi and Tsallis decision trees. However, it is unfeasible for two-parameter fractional Tsallis trees. From a database representation as a complex network, it was possible to determine a set of values for parameters *q*, α, and β based on this network. The experimental results on twelve databases show that the proposed values yield better classifications (AUROC, MCC) for eight of them, and for the four remaining, the classification was equal to that produced by classical trees.

Moreover, two values ($q_{alpha^{\prime }}$, $q_{beta^{\prime }}$) were tested in Renyi and Tsallis decision trees. The results show that Renyi outperforms the classical trees in four (AUROC) and five (MCC) out of twelve databases. Similarly, Tsallis decision trees produced better classification for five (AUROC) and four (MCC) databases. The classification was worse in almost three and one databases for Renyi and Tsallis, respectively. The overall results of both parametric decision trees suggest that both outperform the classical trees in seven databases. All of the above is less favorable than what happened in eight databases analyzed with the two-parameter fractional Tsallis decision trees. In addition, the databases with a better classification using Tsallis decision trees are a subset of those for which two-parameter fractional Tsallis trees produced a better classification. It supports the conjecture that two-parameter fractional Tsallis entropy is a finer measure than the parametric entropies such as Renyi and Tsallis.

The approximate technique for the tree parameters introduced here is a valuable alternative for practitioners. Furthermore, the network classification based on the non-extensive properties of Tsallis and two-parameter fractional Tsallis entropies reveals that the relationships between the records and their attribute values (modeled by a network) are complex. Such complex relationships are better measured by two-parameter fractional Tsallis entropy, the cornerstone of the proposed decision tree.

The results pave the way for using the two-parameter Tsallis fractional entropy in other data mining techniques such as K-means, generic MST, Kruskal MST, and algorithms for dimension reduction in the future. Our research has the limitation that the databases used in the experiments are not large enough to reveal the reduction in time compared with the trial-and-error approach to set the tree parameters. However, we may conjecture that our method works in large databases, which will be the scope of future research.

## Figures and Tables

**Table 1:** The results of $N_{b}\left(l\right)$ and δ from the network of Figure 3, and the “pseudo matrix” of $\alpha^{\prime }$.

*l*	$N_{b}\left(l\right)$	δ	$\alpha_{l,1}^{\prime }$	$\alpha_{l,2}^{\prime }$	$\alpha_{l,3}^{\prime }$
1	6	-	-	-	-
2	3	0.107	$\alpha_{2,1}^{\prime }$	$\alpha_{2,2}^{\prime }$	$\alpha_{2,3}^{\prime }$
3	2	0.107	$\alpha_{3,1}^{\prime }$	$\alpha_{3,2}^{\prime }$	-
4	2	0.143	$\alpha_{4,1}^{\prime }$	$\alpha_{4,2}^{\prime }$	-
5	1	-	-	-	-

**Table 2:** Database and network features. N = nominal, U = numerical, M = mixed.

Database	Records	Attributes	Type	Classes	Balanced	Nodes	Edges
Breast Cancer	699	9	N	2	No	737	1276
Car	1728	6	N	4	Yes	25	70
Cmc	1473	9	M	3	No	74	264
Glass	214	10	U	7	No	1159	1743
Haberman	306	3	U	2	No	94	395
Hayes	160	5	N	3	No	150	186
Image	2310	19	U	7	Yes	12,705	24,411
Letter	20,000	16	U	16	Yes	282	2700
Scale	625	4	N	3	No	23	90
Vehicle	946	18	U	4	Yes	1434	8064
Wine	178	13	U	3	No	1279	2239
Yeast	1484	9	M	10	No	1917	4907

**Table 3:** The parameters of the fractional Tsallis decision tree were obtained using the networks from discretized databases.

Database	$<q_{\alpha^{\prime }}>$	$<q_{\beta^{\prime }}>$	$<\alpha^{1}>$	$<\beta^{1}>$	$<\alpha^{2}>$	$<\beta^{2}>$
Breast Cancer	0.173	0.189	1.147	1.134	0.853	0.866
Car	0.303	0.347	1.137	1.120	0.863	0.880
Cmc	0.169	0.185	1.152	1.138	0.848	0.862
Glass	0.171	0.187	1.154	1.141	0.846	0.859
Haberman	0.344	0.420	1.333	1.273	0.667	0.727
Hayes	0.269	0.310	1.231	1.200	0.769	0.800
Image	0.117	0.123	1.056	1.054	0.944	0.946
Letter	0.155	0.165	1.05	1.047	0.950	0.953
Scale	0.352	0.421	1.217	1.182	0.783	0.818
Vehicle	0.092	0.096	1.106	1.101	0.894	0.899
Wine	0.119	0.127	1.147	1.138	0.853	0.862
Yeast	4.574	5.081	1.003	1.003	0.997	0.997

**Table 4:** The AUROC and MCC of classical (CT) and two-parameter fractional Tsallis decision trees (TFTT) and their parameters *q*, α, β. + means that AUROC or MCC is statistically greater than AUROC or MCC of CT.

Database	${\mathrm{CT}}_{\mathrm{AUROC}}$	${\mathrm{TFTT}}_{\mathrm{AUROC}}$	${\mathrm{CT}}_{\mathrm{MCC}}$	${\mathrm{TFTT}}_{\mathrm{MCC}}$	*q*	α	β	Param. Set
Breast Cancer	0.959	0.964 ${}^{+}$	0.889	0.967 ${}^{+}$	0.173	0.853	0.866	$<q_{\alpha^{\prime }}>$, $<\alpha^{2}>$, $<\beta^{2}>$
Car	0.981	0.982	0.892	0.912 ${}^{+}$	0.347	0.863	0.880	$<q_{\beta^{\prime }}>$, $<\alpha^{2}>$, $<\beta^{2}>$
Cmc	0.691	0.714 ${}^{+}$	0.315	0.349 ${}^{+}$	0.169	1.152	1.138	$<q_{\alpha^{\prime }}>$, $<\alpha^{1}>$, $<\beta^{1}>$
Glass	0.794	0.874 ${}^{+}$	0.56	0.673 ${}^{+}$	0.171	1.154	1.141	$<q_{\alpha^{\prime }}>$, $<\alpha^{1}>$, $<\beta^{1}>$
Haberman	0.579	0.610 ${}^{+}$	0.18	0.156	0.344	1.333	1.273	$<q_{\alpha^{\prime }}>$, $<\alpha^{1}>$, $<\beta^{1}>$
Hayes	0.869	0.895 ${}^{+}$	0.578	0.645 ${}^{+}$	0.269	1.231	1.200	$<q_{\alpha^{\prime }}>$, $<\alpha^{1}>$, $<\beta^{1}>$
Image	0.994	0.992	0.982	0.978	0.123	1.056	1.054	$<q_{\beta^{\prime }}>$, $<\alpha^{1}>$, $<\beta^{1}>$
Letter	0.969	0.974 ${}^{+}$	0.912	0.934 ${}^{+}$	0.155	0.950	0.953	$<q_{\alpha^{\prime }}>$, $<\alpha^{2}>$, $<\beta^{2}>$
Scale	0.845	0.861 ${}^{+}$	0.678	0.703 ${}^{+}$	0.421	1.217	1.182	$<q_{\beta^{\prime }}>$, $<\alpha^{1}>$, $<\beta^{1}>$
Vehicle	0.762	0.755	0.395	0.387	0.092	0.894	0.899	$<q_{\alpha^{\prime }}>$, $<\alpha^{2}>$, $<\beta^{2}>$
Wine	0.968	0.977 ${}^{+}$	0.933	0.957 ${}^{+}$	0.119	1.147	1.138	$<q_{\alpha^{\prime }}>$, $<\alpha^{1}>$, $<\beta^{1}>$
Yeast	0.743	0.733	0.462	0.463	4.574	0.997	0.997	$<q_{\alpha^{\prime }}>$, $<\alpha^{2}>$, $<\beta^{2}>$

**Table 5:** AUROC and MCC of classical (CT), Renyi (RT), and Tsallis (TT) decision trees. + means that AUROC is statistically greater than AUROC or MCC of CT, and − means the opposite.

Database	${\mathrm{CT}}_{\mathrm{AUROC}}$	${\mathrm{RT}}_{\mathrm{AUROC}}$	${\mathrm{TT}}_{\mathrm{AUROC}}$	${\mathrm{CT}}_{\mathrm{MCC}}$	${\mathrm{RT}}_{\mathrm{MCC}}$	${\mathrm{TT}}_{\mathrm{MCC}}$	$q_{r}$	$q_{t}$
Breast Cancer	0.959	0.971 ${}^{+}$	0.963	0.889	0.901 ${}^{+}$	0.887	$<q_{\alpha^{\prime }}>\;=\;$0.173	$<q_{\alpha^{\prime }}>\;=\;$0.173
Car	0.981	0.983	0.982	0.892	0.906 ${}^{+}$	0.912 ${}^{+}$	$<q_{\alpha^{\prime }}>\;=\;$0.303	$<q_{\beta^{\prime }}>\;=\;$0.347
Cmc	0.691	0.676 ${}^{-}$	0.712 ${}^{+}$	0.315	0.256 ${}^{-}$	0.35 ${}^{+}$	$<q_{\beta^{\prime }}>\;=\;$0.185	$<q_{\alpha^{\prime }}>\;=\;0.169$
Glass	0.794	0.838 ${}^{+}$	0.835 ${}^{+}$	0.56	0.622 ${}^{+}$	0.599 ${}^{+}$	$<q_{\beta^{\prime }}>\;=\;$0.187	$<q_{\alpha^{\prime }}>\;=\;$0.171
Haberman	0.579	0.500 ${}^{-}$	0.610 ${}^{+}$	0.18	0.024 ${}^{-}$	0.152	$<q_{\alpha^{\prime }}>\;=\;$0.344	$<q_{\alpha^{\prime }}>\;=\;$0.334
Hayes	0.869	0.869	0.895 ${}^{+}$	0.578	0.579	0.587	$<q_{\alpha^{\prime }}>\;=\;$0.269	$<q_{\alpha^{\prime }}>\;=\;$0.269
Image	0.994	0.997	0.995	0.982	0.984	0.978	$<q_{\alpha^{\prime }}>\;=\;$0.117	$<q_{\beta^{\prime }}>\;=\;$0.123
Letter	0.969	0.980 ${}^{+}$	0.967	0.912	0.939 ${}^{+}$	0.913	$<q_{\beta^{\prime }}>\;=\;$0.165	$<q_{\alpha^{\prime }}>\;=\;$0.155
Scale	0.845	0.839	0.857	0.678	0.651	0.706 ${}^{+}$	$<q_{\beta^{\prime }}>\;=\;$0.421	$<q_{\beta^{\prime }}>\;=\;$0.421
Vehicle	0.762	0.776	0.748	0.395	0.297 ${}^{-}$	0.371	$<q_{\beta^{\prime }}>\;=\;$0.096	$<q_{\alpha^{\prime }}>\;=\;$0.092
Wine	0.968	0.963	0.976 ${}^{+}$	0.933	0.923	0.924	$<q_{\alpha^{\prime }}>\;=\;$0.119	$<q_{\alpha^{\prime }}>\;=\;$0.119
Yeast	0.743	0.789 ${}^{+}$	0.578 ${}^{-}$	0.462	0.505 ${}^{+}$	0.098 ${}^{-}$	$<q_{\beta^{\prime }}>\;=\;$5.081	$<q_{\alpha^{\prime }}>\;=\;$4.574

**Table 6:** AUROC and MCC of Gini decision trees (GT) and two-parameter fractional Tsallis decision trees (TFTT). + means that AUROC is statistically greater than AUROC or MCC of GT.

Database	${\mathrm{GT}}_{\mathrm{AUROC}}$	${\mathrm{TFTT}}_{\mathrm{AUROC}}$	${\mathrm{GT}}_{\mathrm{MCC}}$	${\mathrm{TFTT}}_{\mathrm{MCC}}$
Breast Cancer	0.963	0.964	0.888	0.967 ${}^{+}$
Car	0.981	0.982	0.897	0.912 ${}^{+}$
Cmc	0.58	0.714 ${}^{+}$	0.357	0.349
Glass	0.712	0.874 ${}^{+}$	0.437	0.673 ${}^{+}$
Haberman	0.52	0.61 ${}^{+}$	0.068	0.156 ${}^{+}$
Hayes	0.871	0.895 ${}^{+}$	0.655	0.645
Image	0.988	0.992	0.946	0.978 ${}^{+}$
Letter	0.962	0.974 ${}^{+}$	0.894	0.934 ${}^{+}$
Scale	0.866	0.861	0.654	0.703 ${}^{+}$
Vehicle	0.71	0.755 ${}^{+}$	0.294	0.387 ${}^{+}$
Wine	0.932	0.977	0.847	0.957 ${}^{+}$
Yeast	0.728	0.733	0.414	0.463 ${}^{+}$

## Data Availability

The data that support the findings of this study are available from the corresponding author upon reasonable request.

## Abbreviations

MDPI	Multidisciplinary Digital Publishing Institute
DOAJ	Directory of open-access journals
TLA	Three-letter acronym
LD	Linear dichroism
